# Fall Risk Assessment Tools for Elderly Living in the Community: Can We Do Better?

**DOI:** 10.1371/journal.pone.0146247

**Published:** 2015-12-30

**Authors:** Pierpaolo Palumbo, Luca Palmerini, Stefania Bandinelli, Lorenzo Chiari

**Affiliations:** 1 Department of Electrical, Electronic, and Information Engineering “Guglielmo Marconi”–DEI, University of Bologna, Bologna, Italy; 2 Geriatric Unit, Azienda Sanitaria di Firenze, Florence, Italy; National Institute for Viral Disease Control and Prevention, CDC, China, CHINA

## Abstract

**Background:**

Falls are a common, serious threat to the health and self-confidence of the elderly. Assessment of fall risk is an important aspect of effective fall prevention programs.

**Objectives and methods:**

In order to test whether it is possible to outperform current prognostic tools for falls, we analyzed 1010 variables pertaining to mobility collected from 976 elderly subjects (InCHIANTI study). We trained and validated a data-driven model that issues probabilistic predictions about future falls. We benchmarked the model against other fall risk indicators: history of falls, gait speed, Short Physical Performance Battery (*Guralnik et al*. 1994), and the literature-based fall risk assessment tool FRAT-up (*Cattelani et al*. 2015). Parsimony in the number of variables included in a tool is often considered a proxy for ease of administration. We studied how constraints on the number of variables affect predictive accuracy.

**Results:**

The proposed model and FRAT-up both attained the same discriminative ability; the area under the Receiver Operating Characteristic (ROC) curve (AUC) for multiple falls was 0.71. They outperformed the other risk scores, which reported AUCs for multiple falls between 0.64 and 0.65. Thus, it appears that both data-driven and literature-based approaches are better at estimating fall risk than commonly used fall risk indicators. The accuracy–parsimony analysis revealed that tools with a small number of predictors (~1–5) were suboptimal. Increasing the number of variables improved the predictive accuracy, reaching a plateau at ~20–30, which we can consider as the best trade-off between accuracy and parsimony. Obtaining the values of these ~20–30 variables does not compromise usability, since they are usually available in comprehensive geriatric assessments.

## Introduction

Falls are particularly common and burdensome among the elderly. About one third of the older population experiences at least one fall each year [1]. Worldwide, it is estimated that falls are responsible for 35 million disability-adjusted life years [2].

The most widely accepted paradigm for fall prevention in community-dwelling older adults consists of three sequential stages: screening for high fall risk, assessment of multiple risk factors for those at high risk, and implementation of a tailored intervention [3]. The initial screening protocol serves to focus time and financial resources on those subjects at increased risk, and to spare low-risk subjects unnecessary inconvenience. The protocol should be short and easy to administer. The subsequent multifactorial assessment is intended to identify the risk factors to be targeted by the intervention.

Medical societies and health authorities have issued guidelines for screening community-dwelling older people for fall risk. The guidelines from the American and British Geriatric Societies (AGS/BGS update 2011) and the English National Institute for Health and Care Excellence (NICE) propose a combination of simple questions about history of falls in the previous twelve months and difficulties in walking or balance, possibly followed by simple functional tests assessing gait and balance (e.g. Timed Up and Go test (TUG), Performance-Oriented Mobility Assessment, and Berg Balance Test) [4]. The US Centers for Disease Control and Prevention (CDC) combine similar questions and functional tests with a questionnaire (their ‘*Stay Independent*’ brochure), which also asks about walking aid use, fear of falling, muscle weakness, proprioception at feet, medications, and depression [5,6]. A previous version of the AGS/BGS guidelines was tested on older disabled women and in community-dwelling older adults and found to be suboptimal with respect to other screening tool and of moderate clinical utility [7]. To the best of our knowledge, no published article reports on the predictive accuracy of current versions of these screening algorithms.

Many other screening tools have been proposed in the literature [3,8–15]. Few of them have been tested outside the derivation cohort. Among those, the TUG has been judged inadequate in several studies [16–18]. Gait speed is an indicator of health state in geriatric populations [19]. Its prognostic value for future falls has been shown to be equivalent to total time to perform the TUG [20]. The Short Physical Performance Battery (SPPB) is a tool to assess physical performance, commonly included in comprehensive geriatric assessments [21,22]. Its association with falls and injurious falls is documented in [23,24]. Its prognostic performance is not reported. History of falls is a strong risk indicator for future falls [3,25,26], although it alone does not suffice for primary prevention. Finally, FRAT-up is a recently proposed predictive tool which issues the probability of falling at least once within the time span of one year [27]. The parameters of FRAT-up were obtained from a systematic review and meta-analysis about risk factors for falls in community-dwelling older people [25]. A comparison between this literature-based approach and data-driven models has never been made.

The first aim of the present study is to test whether a predictive tool, trained using state-of-the-art statistical learning techniques over an extensive dataset, can outperform current tools for fall risk assessment. We trained and tested a statistical model over a dataset pertaining to mobility in a community-dwelling older population in order to obtain an accurate prediction of the number of future falls that a subject will experience. We tested the model on future falls and benchmarked it against these fall risk indicators: history of falls (expressed as number of falls experienced during the twelve months before the assessment), gait speed (usual pace as measured in a 7m walk test), the SPPB summary score, and FRAT-up.

Predictive tools based on a small number of variables could be preferable, as their administration is generally shorter and easier. However, this objective can clash with the need to have good accuracy in prediction. Both requirements, parsimony and accuracy, should be taken into account in designing a feasible screening tool. The second aim of this study is hence to evaluate the trade-off between the number of variables used within the predictive tool and accuracy of their prediction.

## Methods

### Data

The dataset comes from the InCHIANTI study (ClinicalTrials.gov NCT01331512), an ongoing population-based cohort study about mobility in the elderly. It consists to date of four waves, about three years apart, initiated in 1999. At each wave the subjects are assessed on a number of different domains and asked about falls experienced in the previous twelve months. The study protocol was approved by the ethical committee of the Italian National Institute of Research and Care of Aging and complies with the Declaration of Helsinki. All participants (or their proxies) received a detailed description of the study purpose and procedures, and gave their written informed consent. More details about the study design and its rationale can be found in [28,29].

We define a sample of the dataset as an assessment of a single person at a specific wave *t*. The corresponding outcome of the sample to predict is the number of falls as reported by the person at wave *t* + 1. Since each person has participated in up to four waves, there will be up to four samples (and up to three outcomes) for each person. We excluded samples from subjects younger than 65 at the time of the assessment, and samples without information about future falls (e.g. samples from subjects’ final assessments or from subjects who did not respond at wave *t* + 1). Thus, we obtained 2313 samples from 976 subjects.

Each subject was assessed on 3280 variables. In addition, from these variables we derived 18 others considered of interest in fall risk assessment (e.g. ‘living alone’ was derived from questions about social network, ‘pain’ was obtained from questions about pain in specific body parts). Each variable was manually annotated as either continuous or categorical. We did not consider variables not relevant to the outcome (e.g. date of the home interview, nutritional habits), categorical variables with more than two levels, variables missing more than 50% of their values, and variables where the missing-value-imputation procedure (see section *Model development*) did not converge. Thus, the final dataset consisted of 1010 variables. Table 1 provides an overview of the dataset content before and after the procedure for variable selection.

**Table 1:** Number of variables organized by category before and after the procedure for variable selection.

		Number of variables	
Area	Brief description	Before selection	After selection
Home interview	Mini Mental State Examination (MMSE), activities of daily living (ADL), instrumental activities of daily living (IADL), social network, Centre for Epidemiological Studies Depression Scale (CESD), sleep habits, pain, incontinence, physical activity, falls, fear of falling, shoes, smoking habits	987	167
Clinical visit and disease adjudication	Family and personal clinical history, diagnosed medical conditions	1032	157
Physical exam	FICSIT (Frailty and Injuries Cooperative Studies of Intervention Techniques) balance scale, Purdue Pegboard, Stairs, Repeated Chair Stands, several walking tests, joint range of motion, lower limb muscle power, muscle strength, SPPB summary performance score	602	385
Instrumental exams	ECG, ENG, anthropometric measures, Eco-Color-Doppler, blood pressure, peripheral quantitative computed tomography, bioelectrical impedance analysis	210	111
Laboratory exams	Blood and urine assays	356	103
Medications	Drug classes	93	87

### Model development

A probabilistic forecast expresses predictions as a probability distribution over the quantity of interest [30]. We fitted a model that expresses its predictions as negative binomial distributions over the number of falls that a given subject will experience in the following twelve months. The negative binomial distribution is fully specified by two parameters: mean, *μ*, and dispersion coefficient, *θ*. Its variance is *μ* + *μ* ^2^ /*θ*. As *θ* increases, the variance approaches the mean and the negative binomial distribution approaches a Poisson distribution. Accordingly, low values of *θ* parametrize highly dispersed distributions.

**Fig 1:**
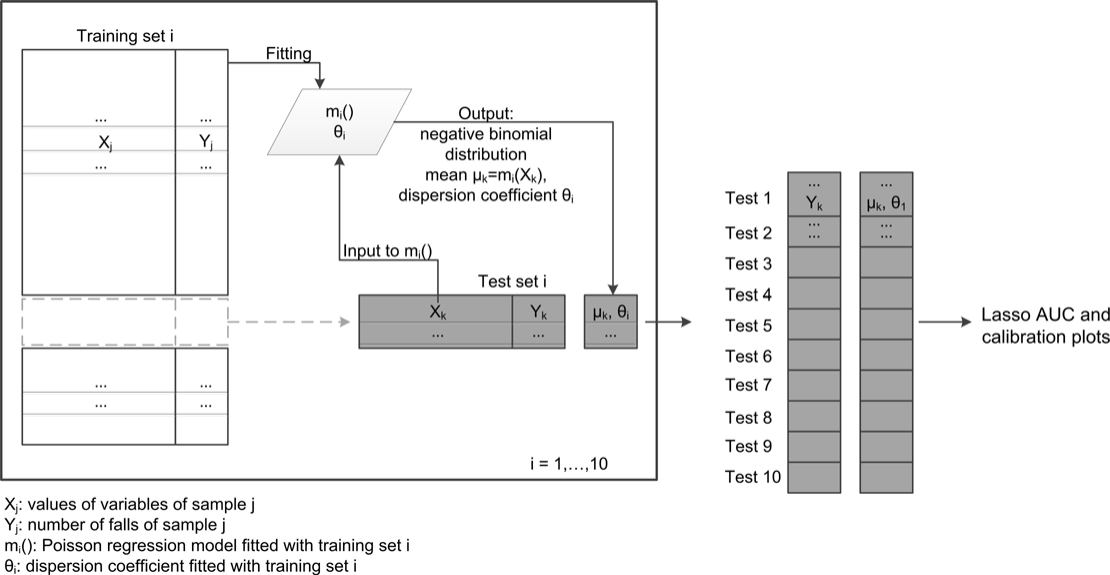
10-fold cross validation scheme. Illustrative scheme of the 10-fold cross validation procedure. During the 10 iterations each sample receives a probabilistic prediction. All these predictions are then used to assess the Lasso model.

We calculated the mean *μ* with the output of a Poisson Lasso (least absolute shrinkage and selection operator) regression model [31,32]. The dispersion coefficient *θ* was calculated from the observed number of falls *Y* and the predictions *μ* issued by the regression model, using maximum likelihood and assuming the number of falls as drawn from a negative binomial distribution with mean equal to *μ* (R function *theta*.*ml* from package *MASS*[33]). Fitting and evaluation were performed with 10-fold cross-validation. Samples were split into 10 folds in such a way that all the samples from the same subject were assigned to the same fold. In turn, nine folds were used to fit a Poisson Lasso regression model [32] and calculate a dispersion coefficient. This regression model and the dispersion coefficient were used to issue the probabilistic predictions on the samples of the test fold (Fig 1). In the following, we refer to this approach as the “unconstrained Lasso model”, or “Lasso model” for brevity.

Each fold used a different imputation model to assign missing data [34]; age, sex, and time to walk 7m at self-selected pace served as predictors of missing values. These three variables were chosen because they are associated with health status.

### Model benchmark

We compared the performance of the trained model against other well-known fall risk indicators: history of falls [3,25,26], gait speed [20], SPPB summary score [21–24], and FRAT-up risk score [27]. History of falls is the number of falls experienced during the twelve months before the assessment, which in the available dataset ranged from zero to a maximum of nine. Gait speed is measured during a 7m walk test, during which the subjects walk at normal pace. The SPPB summary score is the sum of three sub-scores evaluating gait, balance, and sit-to-stand exercises [21,22]. It ranges from zero to twelve, where higher values indicate better performance. FRAT-up is a web-based tool which computes a risk score which expresses the probability, from zero to one, of experiencing at least one fall within twelve months [27]. History of falls, gait speed, and SPPB were already present in the dataset while the FRAT-up score was calculated as in [27].

### Model assessment

We labeled samples (i.e., subjects in a specific wave) as *fallers* if they reported at least one fall at the follow-up after the baseline assessment. Similarly, if they reported more than one fall, they were labeled as *multiple fallers*. All the samples and their associated predictions (history of falls, gait speed, SPPB, FRAT-up, Lasso) were used to evaluate the discriminative ability of the different tools (Fig 1).

We calculated Receiver Operating Characteristic (ROC) curves of the risk scores for fallers and multiple fallers. The ROC curves for the model fitted with Lasso were derived using the means *μ* of the predictive distributions. The discriminative ability was measured as the area under the ROC curve (AUC). The AUC 95% confidence intervals were calculated via the DeLong method [35]. The AUCs were compared with Delong tests for paired ROC curves [35].

The Lasso model was also evaluated for calibration (i.e. the agreement between its predictions and the observed number of falls) by means of a reliability diagram, marginal calibration plot, and probability integral transform (PIT). Reliability diagrams (also known as calibration plots or attribute diagrams) are generally used for dichotomous outcomes [36]. Here the reliability diagram was adapted for count data and used to plot the observed fall rate against the predicted fall rate. The marginal calibration plot shows the observed and predicted number of samples for each possible outcome [37]. PIT is used as diagnostics of probabilistic calibration. It detects whether the variance of the probabilistic predictions agrees with the dispersion of the observations (neutral dispersion), or whether it expresses too little or too much uncertainty (under-dispersion or over-dispersion, respectively) [30]. It was calculated according to the non-randomized procedure for count data described in [37].

### Accuracy-parsimony analysis

The Lasso regression performs variable selection and parameter estimation at the same time. It encourages sparse solutions, i.e. solutions that make use of a small number of variables [31,38]. In order to study how the parsimony of the model affects its predictive accuracy, we studied the performance of the model when fitted under a constraint on the maximum number of variables, *n*, to be included. We refer to this approach as “constrained Lasso model”.

In particular, for a given *n*, we trained the constrained Lasso model according to the 10-fold cross-validation procedure described above. We calculated the mean number of variables actually included in the models averaging over the 10 regression models (one for each fold of the cross-validation) fitted during the cross-validation procedure. We evaluated the accuracy of the model with AUCs for single and multiple fallers, and mean squared error (MSE). MSE was calculated as the mean squared difference between the observed number of falls and the predicted fall rate *μ*. We repeated this analysis varying *n* from 1 to 40.

## Results

The ten unconstrained Lasso models fitted within the procedure of 10-fold cross-validation account for a number of variables that ranges from 21 to 41, with a mean of 29.4. Details about which variables were selected more frequently, and their regression coefficients, are given in S1 Table.

**Fig 2:**
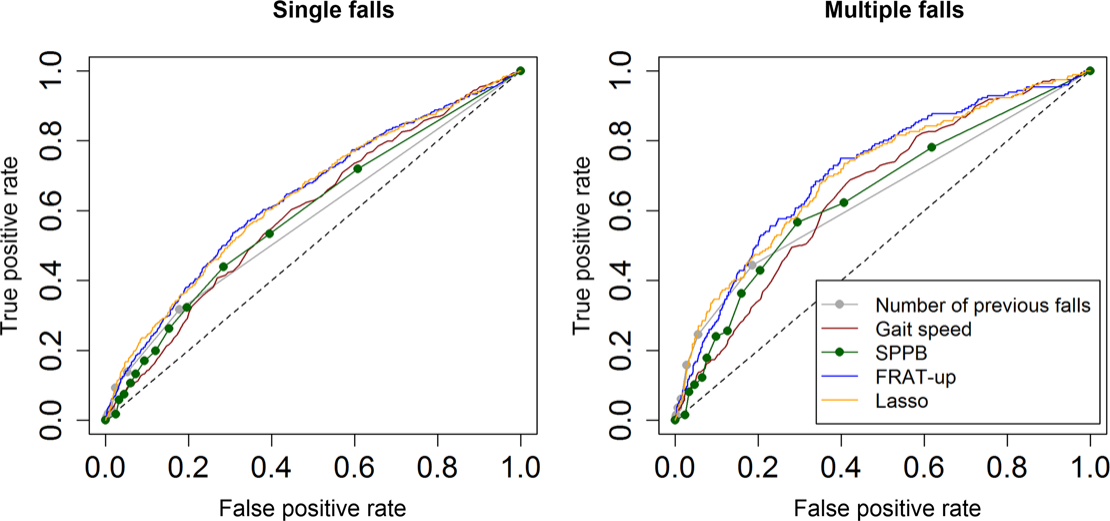
ROC curves. ROC curves of the Lasso model and the other risk indicators (number of previous falls, gait speed, SPPB, and FRAT-up) when predicting for single (left panel) and multiple (right panel) falls.

ROC curves of the five risk scores for single and multiple fallers are shown in Fig 2. The associated AUCs and the results of the hypothesis tests for paired ROC curves are reported in Table 2.

**Table 2:** Discriminative ability of five fall risk indicators. Comparisons with FRAT-up and Lasso model were made with DeLong tests for paired AUCs.

	Single falls		Multiple falls	
	AUC (95% C.I.)	p value. Risk indicator vs FRAT-up / Risk indicator vs Lasso	AUC (95% C.I.)	p value. Risk indicator vs FRAT-up / Risk indicator vs Lasso
**Number of previous falls**	0.574 (0.551–0.597)	** / **	0.640 (0.603–0.678)	** / **
**Gait speed**	0.594 (0.566–0.622)	** / **	0.653 (0.615–0.692)	** / *
**SPPB**	0.590 (0.563–0.618)	** / **	0.645 (0.604–0.686)	** / **
**FRAT-up**	0.638 (0.610–0.666)	– / 0.92	0.713 (0.675–0.752)	– / 0.62
**Lasso**	0.639 (0.611–0.667)	0.92 / –	0.708 (0.669–0.747)	0.62 / –

* = p<0.01

** = p<0.001.

**Fig 3:**
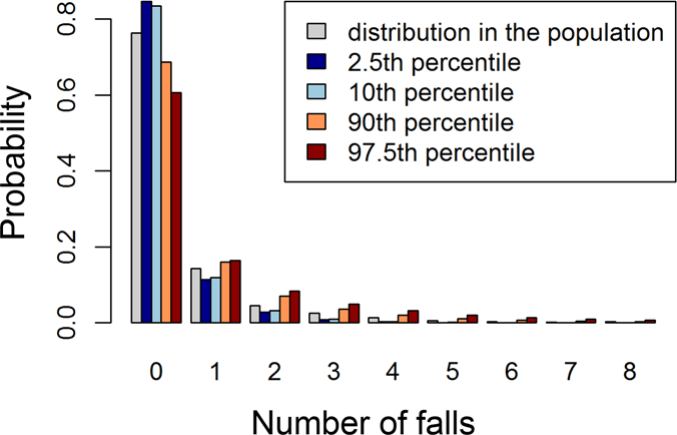
Representative distributions of the Lasso model. Histogram showing the predictive distributions (probability mass functions) on the number of falls for four samples at the 2.5^th^, 10^th^, 90^th^, and 97.5^th^ percentiles of the Lasso risk score. These are compared with the distribution of the number of falls in the InCHIANTI population (in gray).

Fig 3 shows an example of the output of the Lasso model for four representative samples at the 2.5^th^, 10^th^, 90^th^, and 97.5^th^ percentiles of the Lasso risk score, compared with the distribution of the observed number of falls in the InCHIANTI dataset. The expected number of falls for the four selected samples is 0.21, 0.23, 0.66, 1.08, respectively. The fall rate in the InCHIANTI population (baseline data) is 0.42 falls/(person · year).

**Fig 4:**
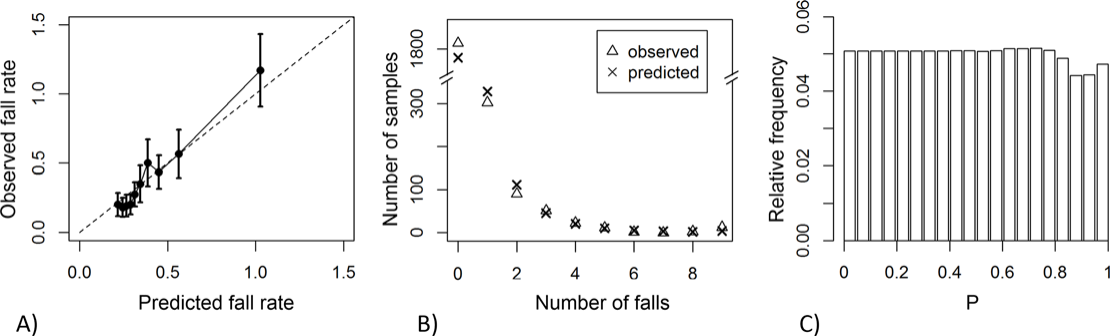
Assessment of Lasso model calibration. (A) Reliability diagram: observed vs predicted fall rate, obtained from grouping samples according to deciles on the risk score; error bars indicate 95% confidence intervals. (B) Marginal calibration plot: observed and predicted number of samples vs number of falls. (C) Histogram of the probability integral transform.

The Lasso model’s reliability diagram, marginal calibration plot, and histogram of the PIT are given in Fig 4. Results for the assessment of marginal calibration are shown in more detail in S2 Table.

**Fig 5:**
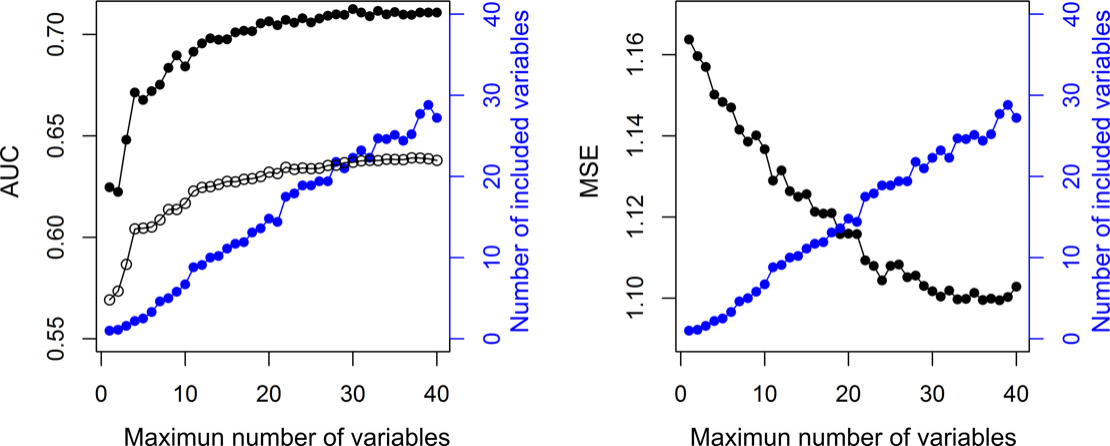
Accuracy-parsimony analysis. Performance of the model when constraining the maximum number of variables to be included in the model. Left panel, left axis: AUC for single falls (black empty circles), AUC for multiple falls (black filled circles). Right panel, left axis: MSE (black filled circles). Both panels, right axes: mean number of variables that were actually included in the models (blue circles).

Fig 5 reports the results of the accuracy-parsimony analysis. The mean number of variables for the ten constrained regression models is less than the maximum number of variables *n* set initially by the constraint. As we relax the constraint (i.e. as *n* increases), the mean number of variables included in the models increases, and the predictive accuracy (measured with MSE and AUC for single and multiple fallers) improves until a plateau is reached. For the AUC this occurs at about 20 variables; for the MSE at about 30 variables.

## Discussion

In this study we have developed a model for fall prediction using a dataset that is large, in terms of number of samples and number of variables related to mobility. We have assessed its predictive properties and benchmarked it against four other risk indicators. We have further investigated whether and to what degree the parsimony of the model compromises its predictive accuracy.

The results show that the AUCs of the Lasso model and FRAT-up are similar and significantly higher than the other risk scores. FRAT-up parameters were derived from the literature [25,27], while the approach proposed here is strongly data-driven. The equivalence of discriminative ability between Lasso and FRAT-up confirms the validity of the literature-based approach.

More analyses presented in S1 File show that the possible hazards of training statistical models in high-dimensional spaces have been avoided. In particular, the learning curves show that more samples for training will not lead to substantial improvements in AUC or MSE. Higher values of AUC could instead be reached from different sources of information, e.g. from variables derived from wearable inertial sensors [12]. AUCs for multiple falls are higher than AUCs for single falls. This result is consistently found for all the risk indicators, and was already reported by other empirical [39] and modeling studies [40].

The Lasso model is well calibrated (Fig 4). Calibration refers to different properties of statistical consistency between predictions and observations [30,41]. The reliability diagram (Fig 4A) shows that the number of predicted falls agrees with the number of observed falls on samples grouped in deciles of the risk score. It also shows that the fall rate is constant across the first 4 deciles (40% of the samples). This represents poor discrimination among those at low risk, since increasing values of risk do not correspond to increasing values of observed falls. The marginal calibration plot (Fig 4B) shows that the model performs fairly well in predicting how many samples will demonstrate a given number of falls (see also S2 Table). The histogram of the PIT (Fig 4C) shows that the model is neutrally dispersed; that is, the negative binomial distributions (meant to express the predictions) have a variance that reflects the right amount of uncertainty about the number of falls that the subject will experience [37]. Conversely, Poisson predictions, obtained without calculating the dispersion coefficient, substantially underestimated the number of non-fallers and exhibited under-dispersion (S1 Fig). Given the unexplained variance in fall incidence across different studies [42], however, the good calibration properties obtained using the InCHIANTI dataset are not guaranteed to hold on other datasets.

Fall risk indicators such as gait speed, SPPB score, and history of falls can be interpreted as performance scores: the lower (or higher, in the case of history of falls) their values, the higher the risk of falling. In contrast, FRAT-up and the Lasso model provide probabilistic predictions. FRAT-up outputs the probability of falling at least once during the twelve months after the assessment; the Lasso model supplies the probability distribution of the number of falls that will be experienced during the same time span. Predicting the number of falls instead of a dichotomous outcome (whether at least one or two falls will occur) provides more information without drawbacks.

Expressing a prediction in probabilistic terms has advantages compared to simpler risk indicators. First, it is possible to aggregate and compare probabilistic predictions issued by different tools for the same health outcome; moreover, the risks of different health outcomes can be compared. Second, calibrated probabilistic models provide accurate statements about groups of subjects. Third, since the prediction is expressed as a probability distribution, its interpretation does not rely on any specific knowledge. In contrast, interpreting other scores requires tool-specific knowledge (e.g. their admissible range and whether they are positively or negatively oriented). Multidisciplinary research is currently investigating how to best express predictions in order to facilitate doctor-patient communication, convey the uncertainty associated with the prediction, and the most influential determinants [43–46]. In this regard, a figure that compares the patient’s issued prediction with reference distributions from the general population (see, e.g., Fig 3) could be a viable option to clearly convey the result of the risk assessment together with its associated uncertainty.

The variables that were selected most frequently in the 10-fold validation procedure (S1 Table) are primarily known risk factors and indicators for fall risk (e.g. history of falls, self-perceived health status, depression, number of medications, and use of drugs acting on the central nervous system) [25]. With reference to the categorization of fall risk factors proposed in [1], most of the selected variables are biological risk factors, and few are behavioral or socioeconomic. None is environmental, since the dataset does not contain information on this kind of risk factors. Other selected variables (e.g. use of the antibacterial quinolone) are unexpected. Indeed, it is known that Lasso accidentally selects ‘noisy’ variables. Although other regression techniques alleviate this problem, they are more computationally demanding and have failed to prove better predictive accuracy in situations of low signal-to-noise ratio [47].

The accuracy-parsimony analysis shows that predictive accuracy improves as the number of variables increases, up to 20–30. This result, in line with the multifactorial etiology of falls, may explain why screening tools employing a very small number of variables have suboptimal performance. The AGS/BGS guidelines suggest a cascade of three to six questions and simple assessments: two or more falls in the previous twelve months, acute fall, self-reported difficulties, and assessed abnormalities in gait and balance. The CDC’s ‘*Stay independent*’ brochure [5] contains twelve questions. Its score is integrated with three questions asked directly by the clinician, and possibly with an assessment of gait, balance, and strength [6]. The FRAT-up questionnaire is made of 28 items, with the possibility of leaving some fields blank because it embeds prevalence information on individual risk factors [48]. Tools that require a high number of input variables can be long and expensive to administer if all the information has to be collected de novo. However, much of the information about fall risk factors is already available to the physician, from the geriatric comprehensive assessment [49]. As a result, a fall risk evaluation integrated into the geriatric comprehensive assessment could provide a good prediction without imposing an additional burden on subjects under assessment or their healthcare professionals.

The InCHIANTI dataset allowed us to make an extensive search on different domains related to mobility and falls in the elderly. However, we have to acknowledge some main limitations of the present study. First, we did not have information about fall hazards in the environment. Second, we excluded all the categorical variables with more than two levels from our analysis. Our choice was driven by the will to avoid a further increase in the number of variables, since fitting a regression model means each categorical variable with *p* levels is replaced with *p* − 1 dummy variables. Indeed, this exclusion criterion may have led to the loss of potentially valuable predictors (e.g. variables for the diagnosis for some diseases, which allow values such as ‘Yes’, ‘No’, and ‘Diagnosis not definite’). Lastly, it must be made clear that in this paper predictions have been validated on falls occurring during a period of one year starting two years after the risk factor assessment. This was due to the study design, as described in section Data. This may be one of the causes of the relatively low values achieved on the AUC.

## Conclusions

We have presented the development and assessment of a tool that issues probabilistic predictions on the number of future falls in a cohort of community-dwelling older subjects. We have trained this model over a dataset that is large, in terms of both number of variables related to mobility and falls and number of samples. We have benchmarked it against other risk indicators. The trained model and FRAT-up outperformed simple fall risk indicators. Despite the breadth of the dataset and the use of state-of-the-art tools of statistical learning, the trained model was not able to reach a better discriminative ability than FRAT-up. This finding supports the validity of the literature-based approach used to develop FRAT-up. Both the data-driven and literature-based approaches are better at estimating fall risk than commonly used fall risk indicators.

The accuracy-parsimony analysis has shown that predictive accuracy improves as the number of variables increases up to 20–30. This suggests that fall prediction is more accurate when based on multiple fall risk factors and indicators; thus simplistic screening tests (three to six variables) are suboptimal in terms of predictive accuracy. Since common risk factors and indicators are already part of geriatric comprehensive assessments, integrating prognostic tools for falls into them could improve the prediction without compromising usability.

## Supporting Information

S1 Fig Probability integral transform for Poisson and negative binomial predictions. Histograms of the probability integral transforms for Poisson (left panel) and negative binomial (right panel) predictions. The U shape for the predictions expressed in terms of Poisson distributions indicates under-dispersion, i.e. understatement of the uncertainty associated with the prediction. The flat shape for the predictions expressed as negative binomial distributions indicates neutral-dispersion.
(TIF)

S1 File Learning curves. Performance of the Lasso prediction as a function of the number of samples used for training.
(DOCX)

S1 Table Variables selected in the Lasso regression. Variables that were selected more frequently in the 10-fold validation procedure and their standardized regression coefficients.
(DOCX)

S2 Table Marginal calibration assessment. Observed and predicted number of samples reporting a given number of falls. *Error* = *predicted* − *observed*. The marginal calibration plot is presented in Fig 4B.
(DOCX)
