# Supplementary figures and images for: Fall Risk Assessment Tools for Elderly Living in the Community: Can We Do Better?
Source: PLoS One. 2015 Dec 30;10(12):e0146247. doi: 10.1371/journal.pone.0146247 (PMC4696849; doi:10.1371/journal.pone.0146247)

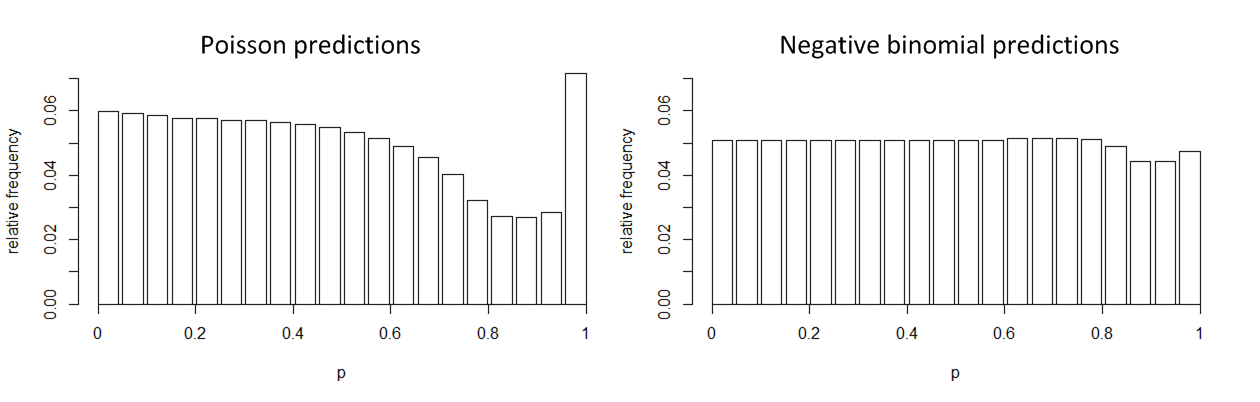

Supplement: S1 Fig — Histograms of the probability integral transforms for Poisson (left panel) and negative binomial (right panel) predictions. The U shape for the predictions expressed in terms of Poisson distributions indicates under-dispersion, i.e. understatement of the uncertainty associated with the prediction. The flat shape for the predictions expressed as negative binomial distributions indicates neutral-dispersion. (TIF) [file pone.0146247.s001.tif]
