# Supplementary material for: Fall Risk Assessment Tools for Elderly Living in the Community: Can We Do Better?
Source: PLoS One. 2015 Dec 30;10(12):e0146247. doi: 10.1371/journal.pone.0146247 (PMC4696849; doi:10.1371/journal.pone.0146247)
Supplement: S1 File — Performance of the Lasso prediction as a function of the number of samples used for training. (DOCX) [file pone.0146247.s002.docx]

# Learning curves

A sensitivity analysis of the performance of the Lasso model to the number of samples available in the training dataset is reported here for model diagnostics.

This analysis is carried out with repeated random sub-sampling validation. The data were split in a training set (percentage $p$ of all the samples) and a test set (percentage $100-p$ of all the samples). For a given percentage $p$, the split was repeated randomly 200 times. Each time, the Lasso model was fitted in the training set and assessed in both the training set and the test set. The performance of the model was taken as the mean of the performance achieved across the 200 random splits. Each split in training and test sets was done so that all the samples relative to one same subject were consistently assigned to the same set.

We took AUC for single and multiple fallers, and mean squared error (MSE) as performance indices.

We repeated this analysis with different values of $p$, from 10% to 90%, obtaining the learning curves shown in Fig. 1 S1 File.


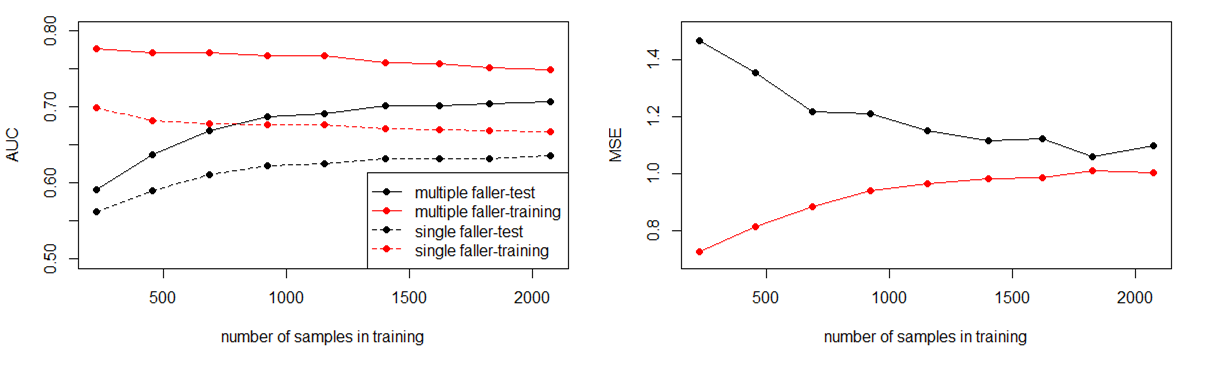


Figure 1 S1 File. Learning curves. Sensibility of the performance of the trained models on the sample size of the training set. Left panel: AUC for single (dots joint by dashed line) and multiple (dots joint by solid line) fallers. Right panel: mean squared error. In red the performances measured in the training set, in black the performances achieved in the test set.

As expected, the performance in the test set improves as the number of samples available for training increases. The curves are close to reach a plateau when the samples available for training are about 2000. Indeed, this is approximately the number of samples used to train the Lasso regression models in the 10-fold cross validation procedure. The learning curves thus suggest that the performance would not increase substantially if we had more samples available for training. We thus think that the choice to train the model over a large space (1010 variables) is supported by the evidence that there is a sufficiently high number of samples for training.
