# Supplementary material for: Fall Risk Assessment Tools for Elderly Living in the Community: Can We Do Better?
Source: PLoS One. 2015 Dec 30;10(12):e0146247. doi: 10.1371/journal.pone.0146247 (PMC4696849; doi:10.1371/journal.pone.0146247)
Supplement: S1 Table — Variables that were selected more frequently in the 10-fold validation procedure and their standardized regression coefficients. (DOCX) [file pone.0146247.s003.docx]

| **Variable** | **Mean standardized regression coefficient** | **Number of times it was selected** |
| --- | --- | --- |
| Number of previous falls | 0.17 | 10 |
| Number of drugs | 0.08 | 10 |
| Self-perceived health status | -0.07 | 10 |
| Previous falls (yes/no) | 0.06 | 10 |
| Drugs for dementia (yes/no) | 0.06 | 10 |
| CESD depressed mood scale (0-28) | 0.04 | 10 |
| Q: “If you are retired, do you have a veteran pension?” (yes/no) | 0.03 | 10 |
| Q: “Can you walk 300 meters twice without stopping?” (yes/no) | -0.03 | 10 |
| Gait speed, 4m usual pace | -0.03 | 6 |
| anti-hypertensive (yes/no) | 0.02 | 7 |
| Q: “Do you have difficulty walking 400 meters on rough terrain?” | 0.02 | 6 |
| Psychoanaleptics: antidepressants (yes/no) | 0.02 | 6 |
| Walking posture: cautious attitude? (yes/no) | 0.02 | 6 |
| Family med hx: siblings diabetic? (yes/no) | 0.02 | 7 |
| Q: “Must you hold onto something (e.g., bannister) while climbing stairs?” (yes/no) | 0.02 | 8 |
| Quinolone antibacterials (yes/no) | 0.02 | 6 |
| Miscellaneous antihypertensives including alpha-blocking agents (yes/no) | 8·10^-6^ | 6 |
