# Supplementary material for: Fall Risk Assessment Tools for Elderly Living in the Community: Can We Do Better?
Source: PLoS One. 2015 Dec 30;10(12):e0146247. doi: 10.1371/journal.pone.0146247 (PMC4696849; doi:10.1371/journal.pone.0146247)
Supplement: S2 Table — Observed and predicted number of samples reporting a given number of falls. Error = predicted − observed. The marginal calibration plot is presented in Fig 4B. (DOCX) [file pone.0146247.s004.docx]

| Number of falls | 0 | 1 | 2 | 3 | 4 | 5 | 6 | 7 | 8 | 9 or more | Total |
| --- | --- | --- | --- | --- | --- | --- | --- | --- | --- | --- | --- |
| Observed | 1814 | 303 | 91 | 52 | 23 | 12 | 2 | 0 | 3 | 13 | 2313 |
| Predicted | 1780.0 | 328.6 | 112.3 | 46.0 | 21.0 | 10.4 | 5.6 | 3.2 | 1.9 | 4.1 |  |
| Error | -34.0 | 25.6 | 21.3 | -6.0 | -1.2 | -1.6 | 3.6 | 3.2 | -1.1 | -8.9 |  |
